# Anti-carbamylated protein antibodies in systemic sclerosis

**DOI:** 10.1186/s13075-023-03099-3

**Published:** 2023-07-21

**Authors:** Sophie I. E. Liem, E. M. Hoekstra, E. W. Nivine Levarht, Annemarie L. Dorjee, Hans U. Scherer, René E. M. Toes, Tom W. J. Huizinga, Jeska K. de Vries-Bouwstra, Cynthia M. Fehres

**Affiliations:** Department of Rheumatology, Leiden University Medical Center, P.O. Box 9600, 2300 RC Leiden, The Netherlands

**Keywords:** Systemic sclerosis, Anti-carbamylated protein antibodies, Skin involvement

## Abstract

**Background:**

To investigate the presence of different isotypes of anti-carbamylated protein (CarP) antibodies in systemic sclerosis (SSc) patients and its association with skin involvement.

**Methods:**

Sera of 194 SSc patients from the Leiden CCISS cohort, fulfilling ACR/EULAR 2013 criteria and a clinical diagnosis of SSc, 83 patients with other connective tissue diseases/Raynaud’s Phenomenon, 24 rheumatoid arthritis patients and 98 age and sex-matched healthy controls were tested for the presence of anti-CarP IgG, IgA and IgM, determined by ELISA. Clinical characteristics, that were evaluated in SSc patients, included age, anti-topoisomerase antibodies (ATA), anti-centromere antibodies (ACA) and modified Rodnan Skin Score (mRSS).

**Results:**

The SSc patients were 55 (SD:13) years and 155 (80%) were female. Forty-four (23%) patients tested positive for ATA, and 80 (42%) ACA. The median mRSS was 2 (range: 0; 47).

Prevalence of anti-CarP IgG was higher in SSc patients than in healthy controls (8% vs 3%, *p* = 0.007. Prevalence of anti-CarP IgA and IgM and levels of anti-CarP isotypes were comparable between SSc patients and healthy controls.

Fifteen (8%) SSc patients tested positive for anti-CarP IgG, 16 (8%) for anti-CarP IgA, and 36 (19%) for anti-CarP IgM. There were no significant correlations between age and levels of anti-CarP isotypes. No correlation between anti-CarP IgG levels and mRSS was found (*r* = 0.141, *p* = 0.049), nor for anti-CarP IgM and IgA levels. Anti-CarP IgA levels were higher in ATA compared to ACA positive SSc patients (ATA: 616 aU/ml [359; 1103]; ACA: 424 aU/ml [300; 673], *p* = 0.015).

**Conclusion:**

SSc patients can test positive for Anti-CarP IgG, IgA and IgM. We do not observe a relevant clinical association between anti-CarP antibody response and skin involvement in SSc.

## Background

Systemic sclerosis (SSc) is a rheumatic autoimmune disease, characterized by vasculopathy and fibrosis of the skin and internal organs including heart, lungs and the gastrointestinal system [1]. The pathogenesis is complex and not fully understood, but it is clear that it involves a dysregulation of the immune system as over 95% of SSc patients harbor antinuclear autoantibodies [2, 3]. The most prevalent antinuclear autoantibodies in SSc are anti-centromere antibodies (ACA) and anti-topoisomerase I antibodies (ATA), which associate with distinct clinical phenotypes [4, 5].

Carbamylation is a post-translational modification of proteins that is practically irreversible and can be triggered by inflammation [6]. Previous studies reported the presence of antibodies against carbamylated proteins (anti-CarP) in systemic sclerosis (SSc). Pecani et al. reported a prevalence of anti-CarP IgG in 5.8% of SSc patients and observed higher levels in SSc patients compared to healthy controls [7]. Recently, Favoino et al. evaluated 124 SSc patients and suggested an inverse correlation between levels of anti-CarP IgG and extent of skin involvement [8]. The mechanisms underlying this observation are unclear, but the authors hypothesized that the carbamylated proteins in the skin absorb the circulating anti-CarP antibodies and consequently neutralize the anti-CarP antibodies resulting in reduced serum levels [8]. Starting from this hypothesis, presence and levels of anti-CarP antibodies might offer potential as biomarker reflecting activity of skin disease and possibly skin remodeling.

As the association between the anti-CarP antibody response and skin has only been studied in one cohort, we therefore set out to replicate these findings in the Leiden prospective CCISS (Comprehensive Care in SSc) cohort. We measured anti-CarP IgG, IgA and IgM, and evaluated prevalence of anti-CarP isotypes and their association with skin involvement in SSc patients.

## Methods

### Study design and patients

One hundred ninety four SSc patients from the prospective Leiden Comprehensive Care in SSc (CCISS) cohort [9], fulfilling ACR/EULAR 2013 SSc criteria [10] and with a clinical diagnosis of SSc were included. In addition, 83 patients with other connective tissue diseases (CTD; including: undifferentiated/mixed CTD or primary and secondary Raynaud’s Phenomenon [RP]), 24 patients with rheumatoid arthritis (RA) and 98 age- and sex-matched healthy controls were included from the Biobank rheumatology*.* All protocols were approved by the Leiden University Medical Center ethics committee (REU 043/SH/sh and REU002e/SH/sh) and patients provided written consent prior to inclusion.

### Anti-CarP assay and measurements

Anti-CarP IgG, IgA and IgM antibodies in the sera from patients and controls were detected by ELISA as previously described [6]. Briefly, Nunc MaxiSorp plates (Thermo Scientific) were coated with 10 μg/ml fetal calf serum (FCS; Bodinco) and carbamylated (Ca)–FCS at 4 °C overnight. The plates were blocked with 1% bovine serum albumin (Sigma) at 4 °C for 6 h, followed by incubation overnight with 1:50 diluted sera on ice. Bound antibodies were detected by incubation for 4 h with horseradish peroxidase–conjugated rabbit anti-human IgG, horseradish peroxidase–conjugated rabbit anti-human IgA or horseradish peroxidase–conjugated rabbit anti-human IgM on ice and subsequently visualized with ABTS. Absorbance was measured at 415 nm and transformed to arbitrary units (AU) per ml using the titration curve of a serum pool from > 5 anti-CarP IgG–positive samples for the anti-CarP IgG ELISA and a pool of 2 anti-CarP IgM and IgA-positive sera for the anti-CarP IgA and IgM ELISA. The background signal of FCS was subtracted from the signal of Ca-FCS to analyse the specific anti-CarP antibody reactivity. The cut-off was equivalent to the mean + 2SD of the healthy controls for each antibody isotype. Samples were considered positive for anti-CarP antibodies if the signal was above the cut-off and the OD of the CarP-FCS signal was at least twice as high as the OD of the background FCS signal.

### Clinical characteristics

For this study, data on clinical characteristics were gathered at the same moment as the sera collection. Sociodemographic data included age, sex, smoking habits (never, current or former). Disease duration was defined by months since first RP and non-RP symptom from the sera collection visit date. Patients were categorized in three autoantibody groups: 1) negative for ATA and ACA, 2) positive for ACA and 3) positive for ATA. One patients was positive for ATA and ACA and excluded from these groups. The extent and severity of skin involvement was measured using the modified Rodnan Skin Score (mRSS) [11]. For the disease subset, patients were categorized as non-cutaneous, limited cutaneous or diffuse cutaneous according to the pattern of skin involvement [12]. Synovitis (yes/no) was evaluated by an experienced rheumatologist.

### Statistical analysis

Descriptive data is presented as means and standard deviations for normally distributed data and median and interquartile range for non-normally distributed data. Anti-CarP isotype levels were compared between the disease groups using Kruskall Wallis tests. If there was a difference found, further exploration between two groups was done with Mann–Whitney-U tests. To evaluate associations between the anti-CarP isotype levels and age, disease duration and mRSS, Spearman correlation tests were used. Proportions were compared using Chi-Square tests. In order to evaluate a possible association between the anti-CarP response and mRSS, we performed a sensitivity analysis in a subgroup of early diffuse cutaneous SSc patients (defined as a non-RP duration of less than 24 months at sera collection). Given the number of analyses performed, Bonferroni correction for multiple testing was applied. A *p*-value of less than 0.05 was considered statistically significant prior to the correction. After the correction, this was adjusted to 0.001, allowing for up to 50 tests. All analyses were conducted using SPSS 25.0 software (SPSS Inc., Chicago, IL, USA).

## Results

### SSc patient characteristics

In total, 194 SSc patients, 83 patients with other CTD/RP, 24 patients with RA and 98 healthy controls were included. The SSc patients were 55 (SD: 13) years and 155 of the 194 (80%) were female. Forty-five (23%) patients tested ATA positive and 81 (42%) ACA positive. The median mRSS was 2 (range: 0; 47) and the mean was 4.7 (SD: 6.9); 36 (19%) patients had non-cutaneous SSc, 119 (61%) limited cutaneous SSc, and 39 (20%) diffuse cutaneous SSc. Twelve (6%) patients had synovitis (Table 1).

**Table 1:** Characteristics of 194 included systemic sclerosis patients

	Total *n* = 194	Diffuse cutaneous SSc *n* = 39	Limited cutaneous SSc *n* = 119	Non-cutaneous SSc *n* = 36
Female, n (%)	155 (80)	26 (64)	98 (82)	32 (89)
Age (years), mean (SD)	55 (13)	56 (14)	54 (14)	56 (13)
RP duration (months), median (IQR)	101 (46; 232)	48 (11; 106)	124 (42; 239)	79 (36; 239)
Non-RP duration (months), median (IQR)	45 (23; 135)	13 (6; 43)	56 (13; 163)	25 (6; 38)
Smoking				
• Current, n (%)	23 (12)	4 (10)	14 (12)	5 (14)
• Former, n (%)	84 (43)	20 (51)	45 (38)	19 (53)
• Never, n (%)	87 (45)	15 (39)	60 (50)	12 (33)
Anti-topoisomerase antibodies, n (%)	44 (23)	18 (46)	25 (21)	2 (6)
Anti-centromere antibodies, n (%)	80 (41)	2 (5)	55 (46)	24 (67)
Modified Rodnan Skin Score, median (IQR)	2 (0; 6)	10 (6; 18)	3 (1; 5)	-
Cardiac involvement, n (%)	7 (4)	3 (8)	2 (2)	2 (6)
Pulmonary arterial hypertension, n (%)	7 (4)	3 (8)	4 (3)	-
ILD on HRCT, n (%)	86 (46)	26 (70)	49 (42)	11 (32)
Gastrointestinal symptoms, n (%)	81 (43)	20 (54)	49 (42)	12 (35)
Calcinosis, n (%)	41 (22)	5 (14)	29 (25)	7 (21)
Digital ulcers, n (%)	20 (11)	2 (5)	16 (14)	2 (6)
Synovitis, n (%)	12 (6)	3 (8)	7 (6)	2 (6)

Data on the clinical characteristics were gathered at the same moment as the sera collection

*N* number, *SD* standard deviation, *RP* Raynaud’s phenomenon, *IQR* interquartile range

### Presence of anti-CarP between the different disease groups

Prevalence of anti-CarP IgG was significantly different between the disease groups (healthy controls: 3%, other CTD/RP: 1%, RA: 25%, SSc: 8%, *p* < 0.001). Further exploration revealed that, prevalence of anti-CarP IgG was significantly higher in RA compared to healthy controls (*p* < 0.001) and compared to other CTD/RP (*p* < 0.001). The prevalence of anti-CarP IgG was higher in SSc compared to healthy controls (8% vs 3%, *p* = 0.007).

**Fig. 1:**
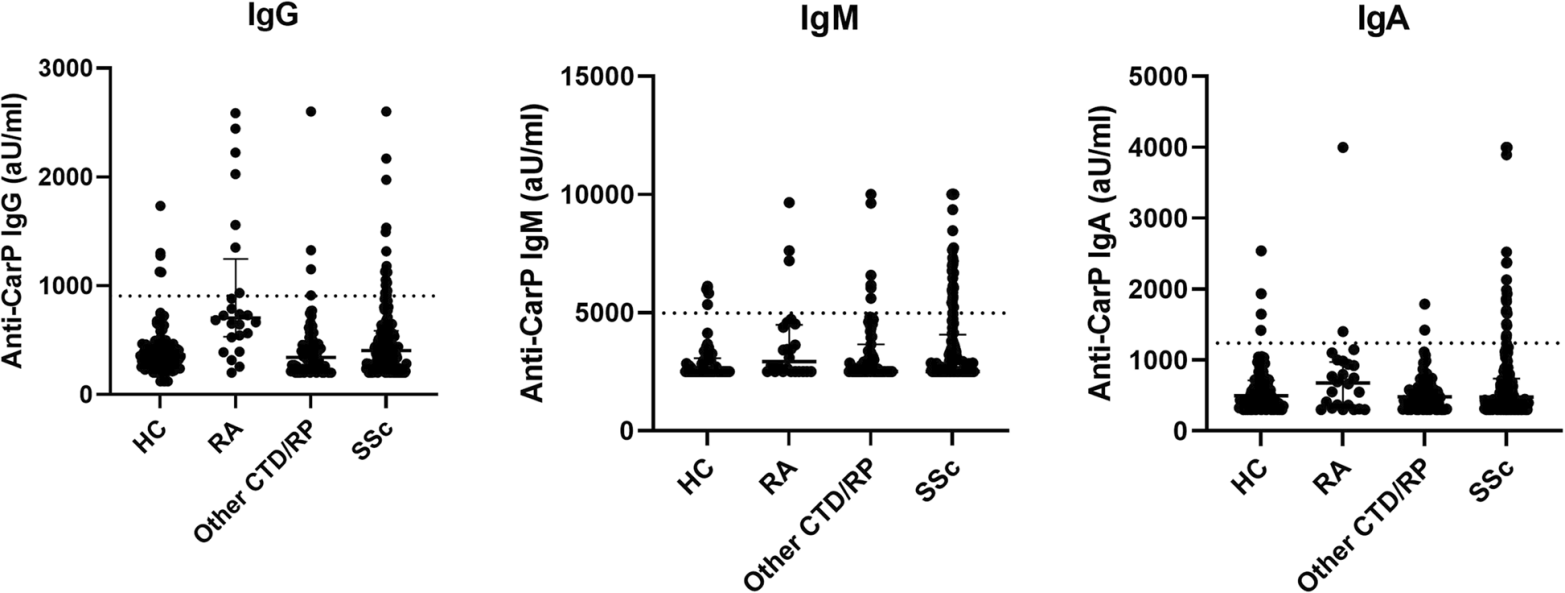
Levels of anti-CarP antibodies isotypes in the different disease groups. HC: healthy controls, RA: rheumatoid arthritis, SSc: systemic sclerosis. Anti-CarP IgG significantly different between healthy controls and rheumatoid arthritis (*p* < 0.001), RA and SSc patients (*p* < 0.001), RA and other CTD/RP, but not between healthy controls and SSc patients (*p* = 0.075). No differences between the disease groups were observed for anti-CarP IgM and IgA

Levels of anti-CarP IgG differed significantly between the disease groups (*p* < 0.001), whereas levels of anti-CarP IgA and IgM were comparable between the groups (Fig. 1). Upon further exploration, IgG levels were significantly different between healthy controls and RA (*p* < 0.001), RA and SSc patients (*p* < 0.001), and RA and other CTD/RP (*p* < 0.001). A trend was observed between healthy controls and SSc patients (*p* = 0.075).

For SSc, the median level of anti-CarP IgG was 404 aU/mL (IQR: 277; 588), of anti-CarP IgM 2510 aU/mL (IQR: 2500; 4063) and of anti-CarP IgA 478 aU/mL (IQR: 317; 737) (Fig. 1 and Table 1). Based on the cut-off values, 15 (8%) of the 194 patients were positive for anti-CarP IgG, 16 (8%) for anti-CarP IgA and 36 (19%) for anti-CarP IgM (Fig. 1). Thirteen patients were positive for two isotypes (*n* = 1 for IgA and IgG, *n* = 7 for IgA and IgM and *n* = 5 for IgG and IgM) and one patient for all three isotypes.

Within the SSc patients, three patients were positive for rheumatoid factor and one patient was positive for both rheumatoid factor and ACPA. The patient both positive for rheumatoid factor and ACPA was positive anti-CarP IgG antibodies, but did not have an overlap disease. Of the three patients positive for rheumatoid factor, one patient was positive for anti-CarP antibodies, and two patients not of whom one had an overlap disease of SSc and mixed connective tissue disease.

### Clinical associations of anti-CarP in SSc patients

#### Age and disease duration

**Fig. 2:**
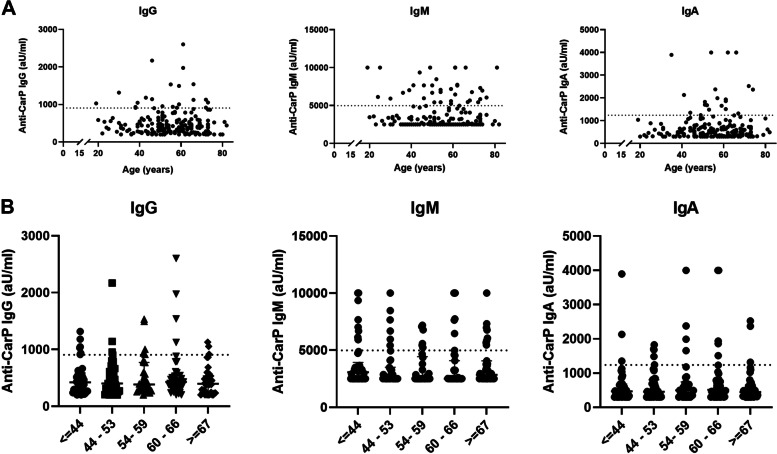
Association between age and anti-CarP isotype levels in SSc patients (*n* = 194). **A** For all isotypes, there were no significant correlations with age (IgG: -0.031, *p* = 0.666, IgM: -0.029 *p* = 0.684, IgA: 0.022, *p* = 0.760). **B** No differences in anti-CarP isotype levels were found between the age groups

First, we evaluated the association between anti-CarP isotype levels and age in the SSc patients by performing a Spearman correlation test. For all isotypes, there were no significant correlations with age (IgG: -0.031, *p* = 0.666, IgM: -0.029, *p* = 0.684, IgA: 0.022, *p* = 0.760; Fig. 2A). Moreover, we divided the SSc patients into five age categories, like the original study of Favoino et al. [8]: I) ≤ 44 years, II) 44 – 53 years, III) 54 – 59 years, IV) 60 – 66 years and V) ≥ 67 years (Fig. 2B). No significant difference in anti-CarP IgG, IgM and IgA between the five groups using Kruskall-Wallis tests were found, nor between group I and group V. No correlation between anti-CarP IgG, IgM or IgA and disease duration was found in the SSc patients (data not shown).

#### Skin involvement

**Fig. 3:**
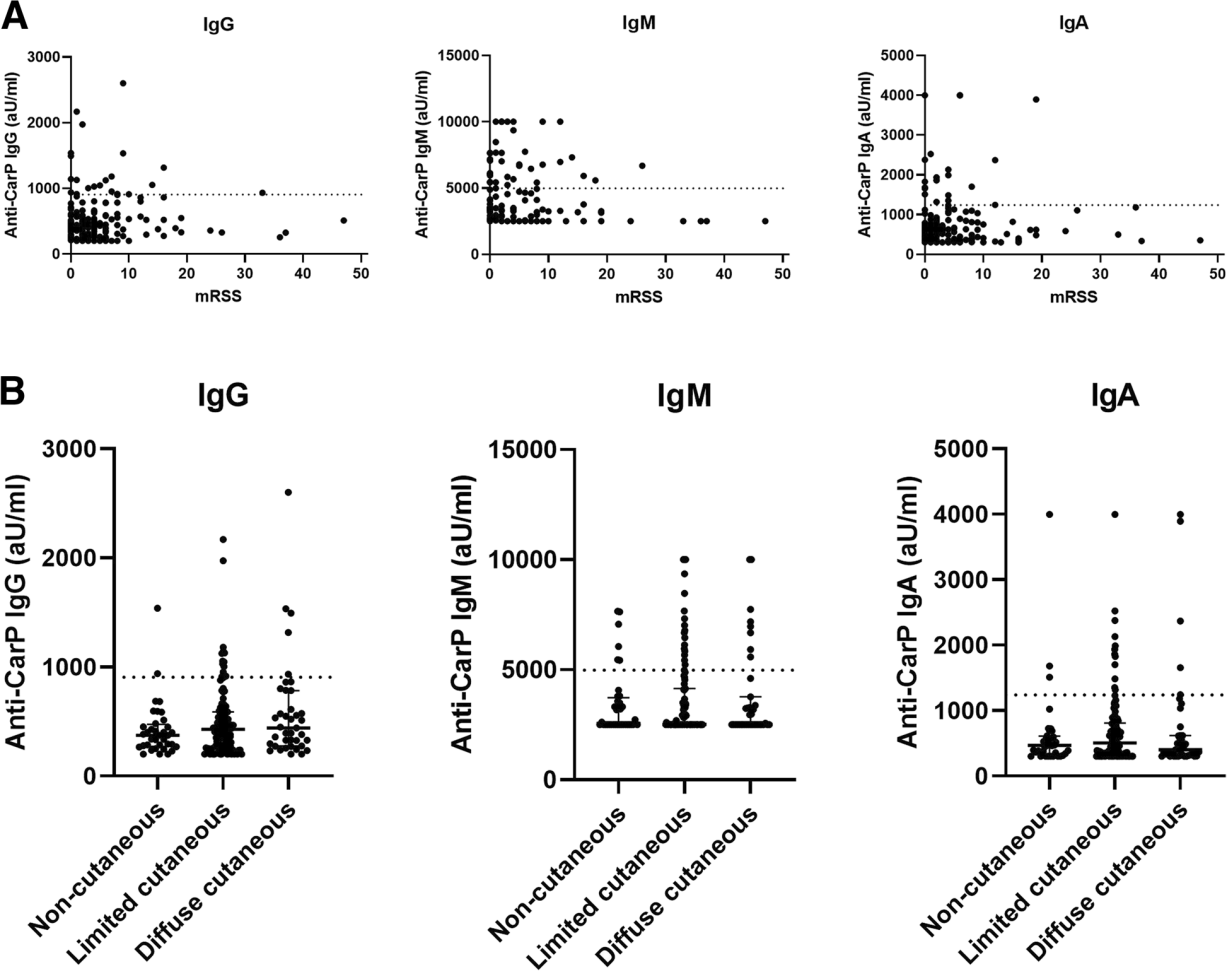
Association between mRSS and disease subset and anti-CarP isotype levels in SSc patients (*n *= 194). A correlation between anti-CarP IgG levels and mRSS in the SSc patients was found (*r* = 0.141, *p* = 0.049), but not for anti-CarP IgM levels (*r* = 0.026, *p* = 0.721) nor for anti-CarP IgA levels (*r* = 0.057, *p* = 0.426). No differences in anti-CarP isotype levels were found between the disease subsets

No association between anti-CarP IgG levels and mRSS in the SSc patients was found (*r* = 0.141, *p* = 0.049), nor for anti-CarP IgM levels (*r* = 0.026, *p* = 0.721) or for anti-CarP IgA levels (*r* = 0.057, *p* = 0.426; Fig. 3a). Anti-CarP IgG positive SSc patients had a higher median mRSS score (7 [IQR: 0; 10]) than anti-CarP IgG negative SSc patients (2 [IQR: 0; 6], *p* = 0.028; Table 2).

**Table 2:** Characteristics in anti-CarP isotype positive and negative SSc patients

	IgG+ *N* = 15	IgG- *N* = 179	IgM+ *N* = 36	IgM- *N* = 158	IgA+ *N* = 16	IgA- *N* = 178
Female, n (%)	13 (87)	142 (80)	31 (86)	124 (79)	10 (63)	145 (82)
Age (years), mean (SD)	52 (16)	55 (13)	55 (15)	55 (13)	57 (10)	55 (14)
Disease duration since non-RP (months), median (IQR)	92 (40; 160)	43 (22; 122)	46 (22; 98)	45 (23; 136)	45 (20; 70)	46 (24; 138)
Disease duration since RP, (months), median (IQR)	116 (61; 239)	98 (45; 232)	85 (40; 223)	112 (48; 239)	64 (32; 163)	110 (48; 232)
Anti-topoisomerase antibodies, n (%)	5 (33)	39 (22)	11 (31)	33 (22)	7 (44)	37 (21)
Anti-centromere antibodies, n (%)	5 (33)	75 (43)	18 (50)	62 (40)	5 (31)	75 (43)
Modified Rodnan Skin Score, median (IQR)	7 (0; 10)	2 (0; 6)	2 (0; 6)	3 (1; 8)	3 (0; 6)	2 (0; 6)
Disease subset, n (%)						
• Non cutaneous	2 (6)	34 (94)	6 (17)	30 (83)	3 (8)	33 (92)
• Limited cutaneous	8 (7)	111 (93)	22 (19)	97 (82)	9 (8)	110 (92)
• Diffuse cutaneous	5 (13)	34 (87)	8 (21)	31 (80)	4 (10)	35 (90)
Synovitis, n (%)	1 (7)	11 (6)	3 (8)	9 (6)	1 (6)	11 (6)

*Anti-CarP* anti-carbamylated protein antibodies, *SSc* systemic sclerosis, *N* number, *SD* standard deviation, *IQR* interquartile range

No differences in disease subset between anti-CarP positive and negative SSc patients were found for IgG, IgM and IgA (Table 2). In addition, anti-CarP levels of all isotypes were comparable between disease subsets (Fig. 3b).

To make sure we did not miss a possible association between anti-CarP response and mRSS, we performed a sensitivity analysis in 22 (56%) early diffuse cutaneous SSc patients. No association between anti-CarP IgG levels and mRSS in the SSc patients was found (*r* = -0.130, *p* = 0.565), nor for anti-CarP IgM levels (*r* = -0.294, *p* = 0.183) or for anti-CarP IgA levels (*r* = -0.003, *p* = 0.990). The mRSS was comparable between patients positive and negative for the different anti-CarP isotypes.

#### Anti-topoisomerase antibodies (ATA) and anti-centromere antibodies (ACA)

**Fig. 4:**
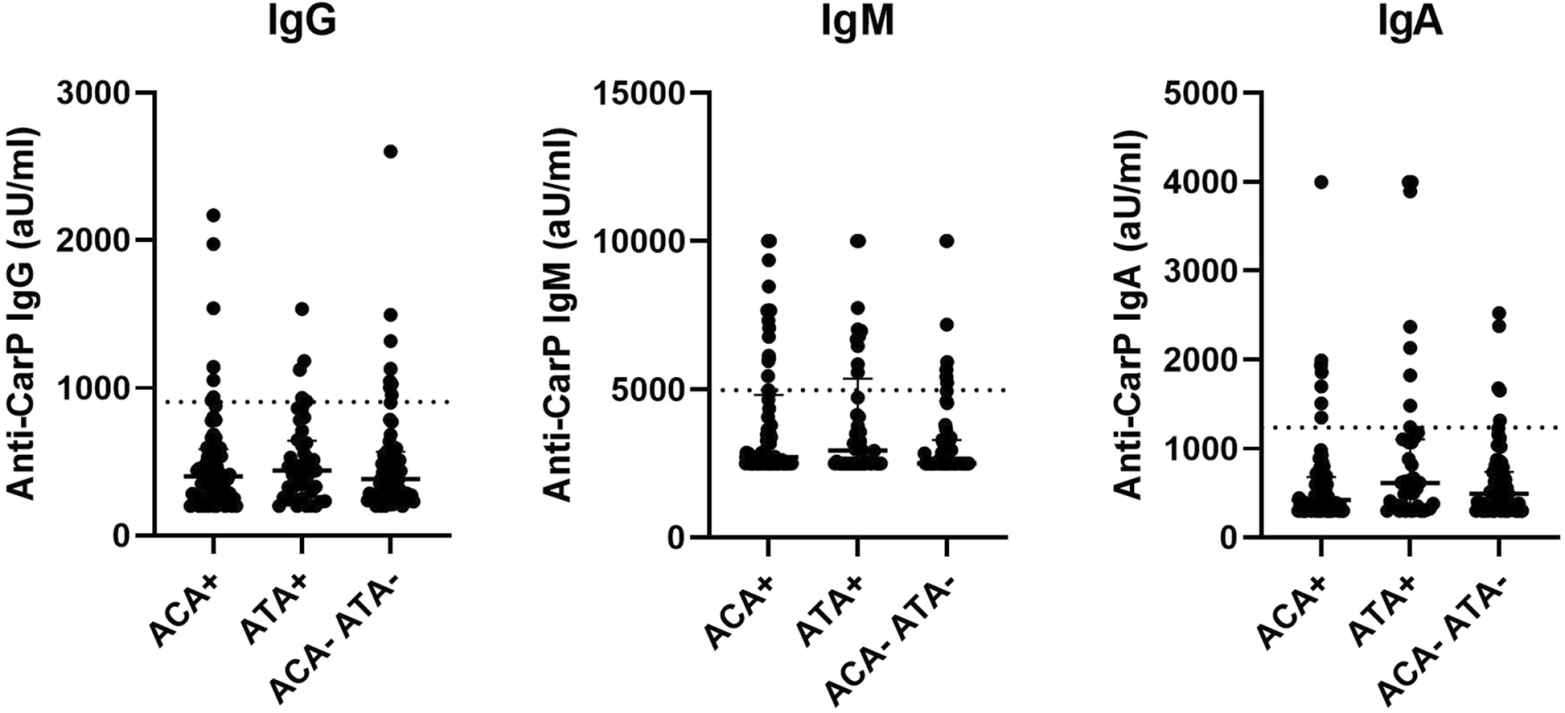
Association between ATA and ACA and anti-CarP isotype levels in SSc patients (*n* = 194). ATA: Anti-topoisomerase I antibodies; ACA: anti-centromere antibodies. A difference in anti-CarP IgA between ATA positive and ACA positive SSc patients (ATA: 616 aU/ml [359; 1103]; ACA: 424 aU/ml [300; 673], *p* = 0.015) was found

With the knowledge that ATA and ACA are established biomarkers for skin involvement and the hypothesis that anti-CarP antibodies might have potential for this, we evaluated the association between anti-CarP antibodies and ATA and ACA positive SSc patients. No difference between prevalence of anti-CarP IgG, IgA or IgM were found between the different autoantibody groups. Levels of anti-CarP IgM and IgA differed slightly between the autoantibody groups (IgM: *p* = 0.030 and IgA: *p* = 0.043; Fig. 4). Anti-CarP IgA levels were higher in ATA compared to ACA positive SSc patients (ATA: 616 aU/ml [359; 1103]; ACA: 424 aU/ml [300; 673], *p* = 0.015).

For anti-CarP IgM levels, further exploration revealed differences between ATA positive and ATA/ACA negative SSc patients (ATA: 2944 aU/ml [2500; 5159] vs ATA/ACA negative: 2500 aU/ml [2500; 3280], *p* = 0.030) and between ACA positive and ATA/ACA negative SSc patients (ACA: 2713 aU/ml [2500; 4767] vs ATA/ACA negative: 2500 aU/ml [2500; 3280], *p* = 0.017).

#### Synovitis

As anti-CarP antibodies are reported to be associated with increased disease activity and with more severe joint damage in RA patients [13, 14], we assessed whether an association between anti-CarP antibodies and synovitis could be observed in SSc. No association between anti-CarP IgG, IgM or IgA prevalence or levels and presence of synovitis was found in the SSc patients (data not shown).

## Discussion

In this study, we show that all anti-CarP isotypes can be detected in SSc, but their prevalence is comparable to healthy controls. We did not find the previously described association between anti-CarP IgG and age or mRSS in SSc.

Previous studies showed conflicting results regarding prevalence of anti-CarP IgG in SSc: Favoino et al. did not find a difference in anti-CarP IgG between patients and controls [8], while Pecani et al. did observe a higher prevalence among SSc patients [7]. We also observed a higher prevalence of the anti-CarP IgG in SSc (SSc: 8% vs HC: 3%) but the difference was not significant after Bonferroni correction. As such, our observation is more in line with observation of Pecani et al. [7]. This might be explained by the fact that in the study of Favoino et al. anti-CarP ratio versus one RA serum was reported [8] instead of reporting “raw” anti-CarP data which we and Pecani et al. did. Another important difference between our study and the study by Favoino et al. is that they used one serum sample from an undefined RA patient and related the anti-CarP levels of all SSc samples as percentage of binding relative to that control patient [8]. As anti-CarP levels fluctuate in individual RA-patients, we cannot calculate the expression levels of the SSc samples in the Favoino study. It makes it therefore difficult to directly compare our results with the results of Favoino et al..

Favoino et al. reported an inverse association between anti-CarP IgG levels and age at observation (Spearman *R* = -0.27, *p* = 0.002) and between anti-CarP IgG and mRSS (*R* = -0.32, *p* < 0.001) [8]. In contrast, we did not observe an association between age and anti-CarP, nor between anti-Carp IgG, IgA or IgM and mRSS. As compared to our study, next to the methodological issues discussed above, the patients included in the study of Favoino et al. were more frequently female (95% vs 80%), had a higher mean mRSS score (8.7 [SD: 8.4] vs 4.7 [SD: 6.9]) [8], whereas age was similar (53.8 [SD:12.4] vs 55 [SD:13] years).

ATA is associated with the fibrotic complications of SSc [5]. However, it is also known that half of the SSc patients do not develop severe skin disease. Therefore, we hypothesized that anti-CarP antibodies could contribute to predicting active skin disease in SSc and evaluated the association between anti-CarP antibodies and ATA and ACA. We did find a difference in anti-CarP IgA between ATA positive and ACA positive SSc patients (ATA: 616 aU/ml [359; 1103]; ACA: 424 aU/ml [300; 673], *p* = 0.015). However, after correction for multiple testing, this difference was no longer statistically significant. It is however noteworthy, because spontaneous secretion of ATA-IgG and, more remarkably, extensive secretion of ATA-IgA in ATA positive SSc patients was observed, while spontaneous ACA-IgA secretion in ACA positive SSc patients was not observed [15]. This ATA-IgA secretion could point towards a potential mucosal trigger of the ATA response, which warrants further research.

Our study has several strengths and limitations. We used a standard ELISA set-up and measured all anti-CarP isotypes. We performed robust statistical analyses using the data of the anti-CarP isotypes levels, and adjusting for multiple testing. Ideally, to evaluate a potential biomarker, the study population should reflect the general SSc population. Our patients were derived from the prospective Leiden CCISS cohort, and all fulfilled ACR/EULAR 2013 criteria. However, the patients were not selected for active (skin) disease and also stable SSc patients with a lower mRSS were included. This might have reduced the discriminative power of our study to evaluate anti-CarP responses as a biomarker. However, a strong biomarker should be able to discriminate between active and stable patients.

## Conclusion

In conclusion, anti-CarP IgG, IgA and IgM antibodies can be detected in SSc patients but in contrast to a previous study we did not find an association between anti-Carp IgG and mRSS. Furthermore, our data do not support the hypothesis that carbamylated proteins in the skin absorb the circulating anti-CarP antibodies and consequently reduce their serum levels in SSc patients. In summary, the present study does not support a prognostic value for anti‐CarP autoantibodies in SSc or its related skin involvement.

## Data Availability

Data is available upon reasonable request and in accordance with the Dutch privacy laws.

## Abbreviations

ACA: Anti-centromere protein autoantibodies
ACR: American College of Rheumatology
ANA: Anti-nuclear autoantibodies
ATA: Anti-topoisomerase I autoantibodies
CARP: Carbamylated protein
CCISS: Comprehensive Care in SSc (Leiden cohort)
CTD: Connective tissue disease
ELISA: Enzyme-linked immunosorbent assay
EULAR: European Alliance of Associations for Rheumatology
FCS: Fetal calf serum
IgA: Immunoglobulin A
IgG: Immunoglobulin G
IgM: Immunoglobulin M
mRSS: Modified Rodnan skin score
RA: Rheumatoid arthritis
RP: Raynaud’s phenomenon
SSc: Systemic sclerosis
